# First long-term detection of paleo-oceanic signature of dust aerosol at the southern marginal area of the Taklimakan Desert

**DOI:** 10.1038/s41598-018-25166-5

**Published:** 2018-04-30

**Authors:** Qi Zhou, Juan Li, Jian Xu, Xiaofei Qin, Congrui Deng, Joshua S. Fu, Qiongzhen Wang, Mijiti Yiming, Kan Huang, Guoshun Zhuang

**Affiliations:** 1 Center for Atmospheric Chemistry Study, Shanghai Key Laboratory of Atmospheric Particle Pollution and Prevention (LAP3), Department of Environmental Science and Engineering, Fudan University, Shanghai, 200433 China; 2 Institute of Atmospheric Sciences, Fudan University, Shanghai, 200433 China; 3 Department of Civil and Environmental Engineering, University of Tennessee, Knoxville, TN 37996 USA; 4 Environmental Science Research & Design Institute of Zhejiang Province, Hangzhou, Zhejiang, 310007 China; 5 Hetian Environmental Monitoring Center, Hetian, 848000 China

## Abstract

We firstly conducted a long-term *in-situ* field measurement at a marginal area (Hotan) of the southern Taklimakan Desert covering all four seasons. Detailed chemical characterization of dust aerosol over Hotan showed several unconventional features, including (1) ubiquity of high Na^+^ and Cl^−^ abundances in the Taklimakan dust aerosol and its Cl^−^/Na^+^ ratio close to seawater; (2) high Ca content in the Taklimakan dust (7.4~8.0%) which was about two times of that in the natural crust; (3) high abundance of soluble sulfate concentrations and strong correlations between sulfate and Na^+^ and Cl^−^ as well as typical mineral tracers such as Al and Ca. Our results collectively indicated that the dust aerosol from the Taklimakan Desert was characterized of evident paelo-oceanic signature as the Taklimakan Desert was found as an ocean in the ancient times from the perspective of paleogeology. It was estimated that primary sources dominated the total abundances of sulfate during the dust seasons while previous climate modeling works had seldom considered the cooling effects of sulfate from the Taklimakan Desert.

## Introduction

Dust aerosols have been continuously concerned globally in the past several decades due to its important role in atmospheric processes and ecological effects. Asian dust storm originating from arid regions and deserts located in China and Mongolia is one of the major components of the ambient aerosol, which brought negative impacts on the air quality of downstream areas over East Asia, including China^1,2^, Japan^3^, and Korea^4^. Asian dust can be transported to the Northern Pacific^5^, even to North America occasionally^6^, making it play an important role in global biogeochemical cycles^7^. It was estimated that around 800 Tg yr^−1^ dust from the Indo-China peninsula and the Chinese mainland could enter the atmosphere, of which 50% was subject to long-range transport over the Pacific Ocean and beyond^6,8^. It has been found that dust can also affect the climate by influencing the absorption and scattering of sunlight radiation directly and by acting as cloud condensation nuclei indirectly^4^. In addition, compared to the Saharan dust, East Asian dust were more absorptive, and its direct radiative forcing was found nearly positive or nil at the top of the atmosphere, suggesting that the East Asian dust can influence the cloud properties not only by acting as cloud condensation nuclei and ice nuclei but also through changing the relative humidity and stability of the atmosphere^9^. Metallic elements of mineral dust particles can be acidized by acidic gases, which results in the mixing between dust and anthropogenic pollutants with enhancement of secondary aerosol products such as sulfate and nitrate^10–12^. This interaction mainly depended on the size distribution and chemical composition of dust particles^13,14^ and may alter the nitrogen and sulfur cycles and the acid/base balance^15,16^.

The Taklimakan Desert is located in the middle of the Tarim Basin (average elevation of 1.1 km), bounded by the Pamir Plateau to the west, the Kunlun Mountain range to the south and the Tian Shan Mountain range to the north. The eastern margin of the Taklimakan Desert is the only low-elevation gateway for low-level dust to flow out^17^. It was estimated that the annual dust emissions originating from this desert could account for around half of the total dust emissions in Asia^18,19^. Compared to some major deserts, the southern Taklimakan Desert was characterized of the highest frequency of dust storm outbreaks^20^ and dust sands with smaller diameter^21,22^. Based on the modeling results simulated by the RAMS/CFORS (Regional Atmospheric Modeling System/Chemical Weather Forecast System) dust model, Uno *et al*.^23^ revealed that the dust concentration in the southern Taklimakan Desert was higher than its northern part. The vertical profile of dust concentrations was relatively uniform as a result of the warmer air crossing the southern part of the desert, and the strong wind shear facilitated the vertical mixing of dust from the planet boundary layer to the free troposphere. Based on the analysis of satellite data^17^, the transport patterns of the Taklimakan dust were identified quite seasonally dependent. In spring, the dust emissions were confined within the lower atmosphere, while in summer and fall, the dust can be lofted to much higher levels. The Taklimakan Desert dust broke through the planetary boundary layer and extended to the upper troposphere over the northern Tibetan Plateau under special weather conditions^24^. The direct radiative forcing induced by dust was simulated to be both negative at the top of the atmosphere (−3 Wm^−2^) and at the surface (−8 Wm^−2^) during a spring Taklimakan dust storm episode and resulted in an overall heating effect in the atmosphere^25^.

However, till now there has been very limited information about the chemical characteristics of dust aerosol originating from the Taklimakan Desert. Wu *et al*.^26^ surmised that the observed sulfate from the Taklimakan Desert was soil-derived. However, that study was based on the analysis of only 11 samples without statistical meaning. This study firstly investigates the chemical characteristics of dust aerosol over a background site at the southern edge of the Taklimakan Desert based on long-term measurements. Some specific features of the dust aerosol from the Taklimakan Desert were revealed in this study. We expect that the results of this study will provide basic chemical information of the Taklimakan dust, which could be helpful for more accurate retrievals of the optical properties of the dust particles and finally the parameterizations in climate models. Due to the nature of long-range transport of dust at the global scale^27^, more detailed physicochemical characterization of the Taklimakan dust is essential for deepening the understanding of the global biogeochemical cycles of dust.

## Methodology

### Dust observational site and sampling

**Figure 1:**
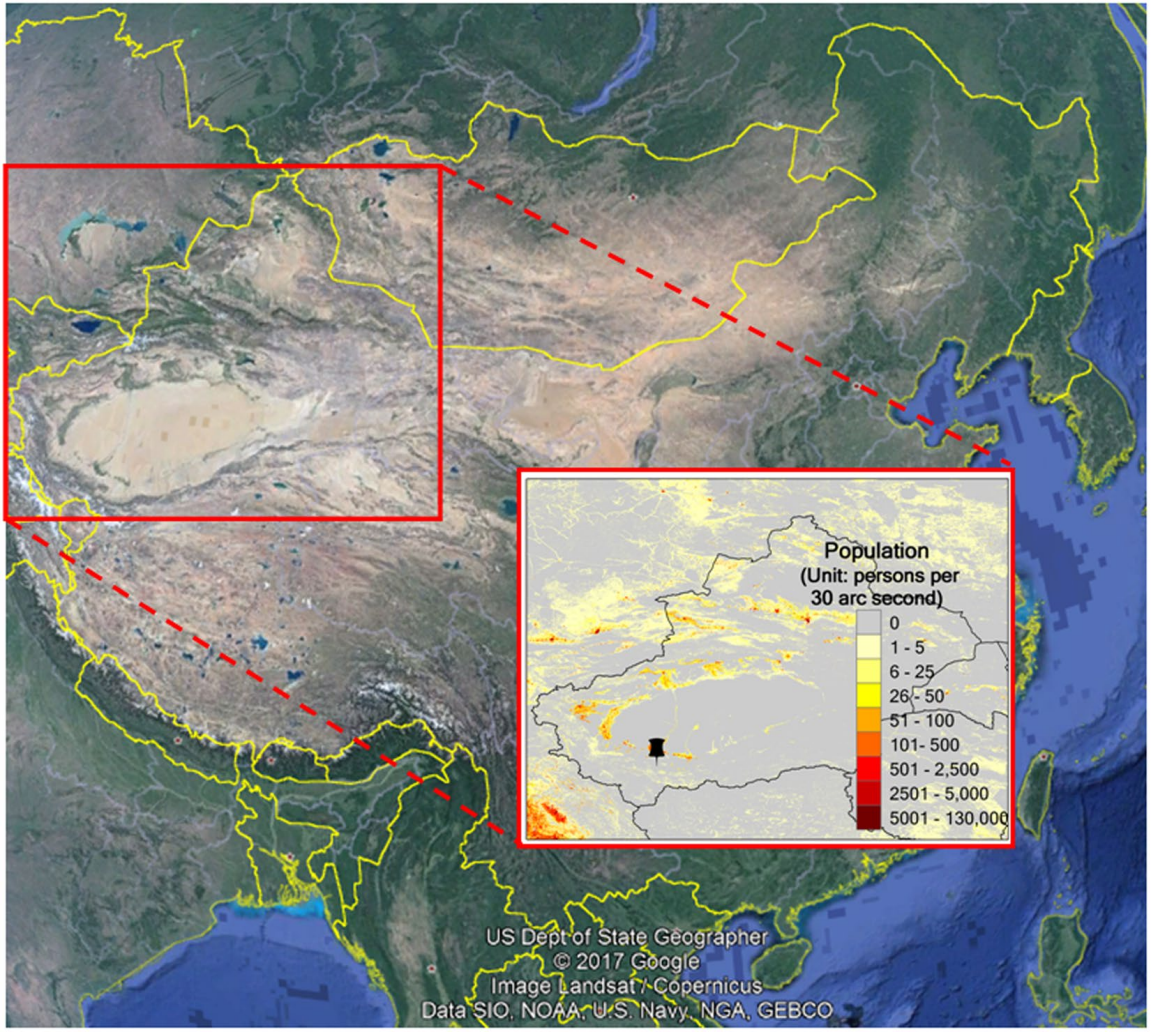
The Hotan observational site set up at the southern Taklimakan Desert is denoted by the black pushpin marker in the enlarged inner figure. Human population density with a spatial resolution of ~1 km^2^ based on the 2010 LandScan global population dataset (http://www.ornl.gov/landscan/) is also plotted (The Asia map is from Google Earth Pro 7.3.0.3832 (32-bit), https://www.google.com/earth/download/gep/agree.html and the population map is created by ArcGIS 10.0, https://www.arcgis.com/features/index.html).

Field measurement of dust aerosol was conducted at Hotan (80.78°E, 37.04°N), an agricultural oasis city at the southern edge of the Taklimakan Desert (Fig. 1). Hotan is located in the arid zone with dry climate with annual precipitation of around 30 mm and annual evaporation of more than 2400 mm. The weather there is windy with more than 200 days of floating dust and around 60 days of dust storms. The monthly dry deposition of dust reached over 100 tons/km^2^. The total area of Hotan is around 580 km^2^ with a human population of 320,000. Its local economy mainly comes from the merchandize sales, tourism, and public transportation. Due to the close geographic location to the Taklimakan Desert and insignificant anthropogenic emissions, Hotan could be regarded as a good representative region for studying the dust aerosol originating from the Taklimakan Desert.

During March, 2011 to January, 2014, TSP (Total Suspended Particles) and PM_2.5_ (fine Particle Matters with a diameter of 2.5 μm or less) samples were collected at the Hotan Meteorological Bureau. We collected the aerosol samples following the China’s National Manual Methods for Ambient Air Quality Monitoring (HJ/T 194–2005). The aerosol samples were collected for around 24 hours (normally from 10:00 to 10:00 LST (Local Standard Time) of the next day) on Whatman 41 filters (Whatman Inc., Maidstone, UK) by using a medium-volume sampler (Beijing Geological Instrument-Dickel Co., Ltd.; model: TSP/PM_10_/PM_2.5_−2; flow rate: 77.59 L min^−1^). The total volumes of air that have been pumped were calculated based on the accurate sampling time and flow rate. Before sampling, the sampler parts that filters were placed on should be rinsed and cleaned. After sampling, the filter was carefully taken off by using a plastic tweezer. All the samples were put in polyethylene plastic bags immediately after sampling and then reserved in a refrigerator. The filters were weighed before and after sampling using an analytical balance (Model: Sartorius 2004MP; reading precision: 10 µg) after stabilizing in constant temperature (20 ± 1 °C) and humidity (40 ± 2%) for 48 hours. All the procedures were strictly quality controlled to avoid the possible contamination of the samples.

### Ion analysis

One-fourth of each sample and the blank filter were extracted ultrasonically by 10 mL deionized water (18 MΩ cm^−1^) for 40 minutes. Four inorganic anions (SO_4_^2−^, NO_3_^−^, Cl^−^, F^−^) and five cations (NH_4_^+^, Ca^2+^, K^+^, Mg^2+^, Na^+^) were analyzed by Ion Chromatography (IC, Dionex ICS 3000, USA), which consists of a separation column (Dionex Ionpac AS11 for Anion, Dionex IonPac CS12A for Cation). The standard deviations of all ions for a repeated six times measurement were less than 5%. The recovery rates of the ions were within 80–120%. Detailed descriptions were given elsewhere^21,28^.

### Element analysis

A quarter of the sample filters were digested at 190 °C for 1 h with 8 mL HNO_3_ and 2 mL HF. After cooling, the solutions were diluted to 30 mL with distilled-deionized water. Blank filters were in parallel processing in order to reduce the error. Total 21 elements (Al, As, Ba, Ca, Cd, Co, Cr, Cu, Fe, K, Mg, Mn, Na, Ni, P, Pb, S, Sn, Sr, Ti and Zn) were determined by ICP-OES (Inductively Coupled Plasma Optical Emission Spectrometer, Model: SPECTRO, Germany). The detailed analytical procedures were given elsewhere^29^.

## Results and Discussion

### Severe atmospheric pollution over Hotan ascribed to frequent dust outbreaks

**Figure 2:**
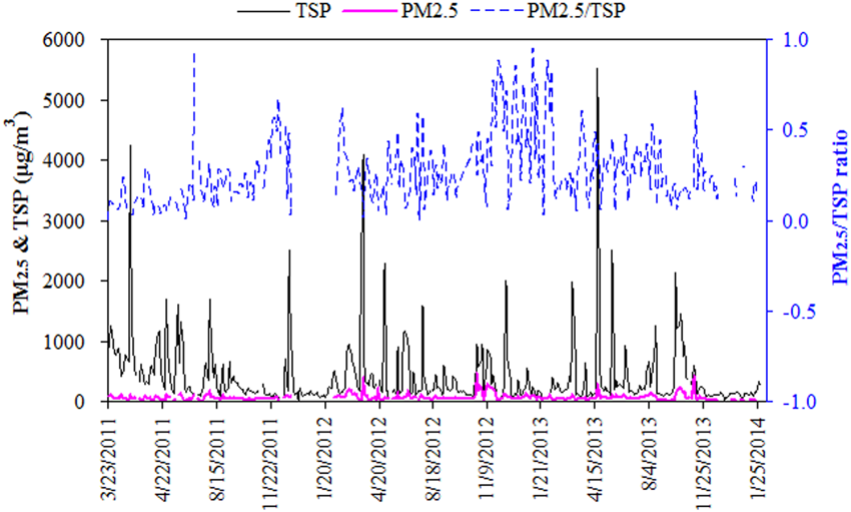
The time-series of TSP and PM_2.5_ over Hotan during 2011–2013 as well as for the mass ratios of PM_2.5_/TSP.

Figure 2 shows the times series of TSP and PM_2.5_ daily concentrations over Hotan from the spring of 2011 to the winter of 2013. The seasonality of TSP over Hotan was obviously intense with the mean monthly concentrations of 653.2 ± 875.8, 416.6 ± 440.9, 326.0 ± 346.9, and 161.0 ± 166.2 μg m^−3^ in spring (March–May), summer (June–August), autumn (September–November), and winter (December – January of the next year), respectively. According to China’s National Ambient Air Quality Standards for TSP (Level II: 300 μg m^−3^), more than one third of the sampling days exceeded this threshold. The maximum daily TSP concentration reached 5522.6 μg m^−3^ in spring, 2514.8 μg m^−3^ in summer, 2135.2 μg m^−3^ in autumn, and 2516.1 μg m^−3^ in winter, respectively. A comparison between Hotan and some typical arid regions is shown in Table S1. Compared to Duolun and Yulin which is located in the Gobi Desert and the Loess Plateau, respectively, Hotan’s TSP concentrations were about 2–3 times higher. As for the fine particles, Hotan showed moderately higher concentrations than Duolun and Yulin. As a result, the mean PM_2.5_/TSP ratio of Hotan was as low as 0.25 ± 0.18 during 2011–2013, significantly lower than the other arid regions shown in Table S1.

We define the days with TSP concentrations higher than 500 μg m^−3^ as the dust storm days according to the Level III daily standard of TSP in China’s National Ambient Air Quality Standard (GB3095–1996). Based on this criterion, about one quarter of a year had been classified as dust storm days, which was relatively consistent with the dust activities of Minfeng County (113.5 days in a year) at the southwestern and southern edges of the Taklimakan Desert^30^.

On average, the concentration of TSP and PM_2.5_ reached 1139.7 ± 868.9 and 114.6 ± 88.5 μg m^−3^ during the dust storm days, respectively, while much lower of 192.5 ± 110.0 and 50.6 ± 32.0 μg m^−3^ during the non-dust storm days. The frequency of dust storm over Hotan in spring, summer, autumn, and winter was around 40%, 25%, 18%, and 6%, respectively. Spring and summer were the seasons characterized of high frequencies of dust storm outbreaks. In winter, the dust storm activities were much weaker due to the low wind speed and hardening soil. This was consistent with the monitoring results that dust depositions exhibited high values from March to August in Cele of Hotan^31^. Hence, we define spring, summer and fall as the dust seasons, and winter as the non-dust season.

Overall, Hotan could be regarded as one of the most serious coarse particle pollution regions in China. At the same time, its fine particle pollution was not negligible, neither. This suggested that the air quality of Hotan has been facing enormous challenges.

### Unique characteristic of dust aerosol over Hotan

#### High sodium and chloride

**Figure 3:**
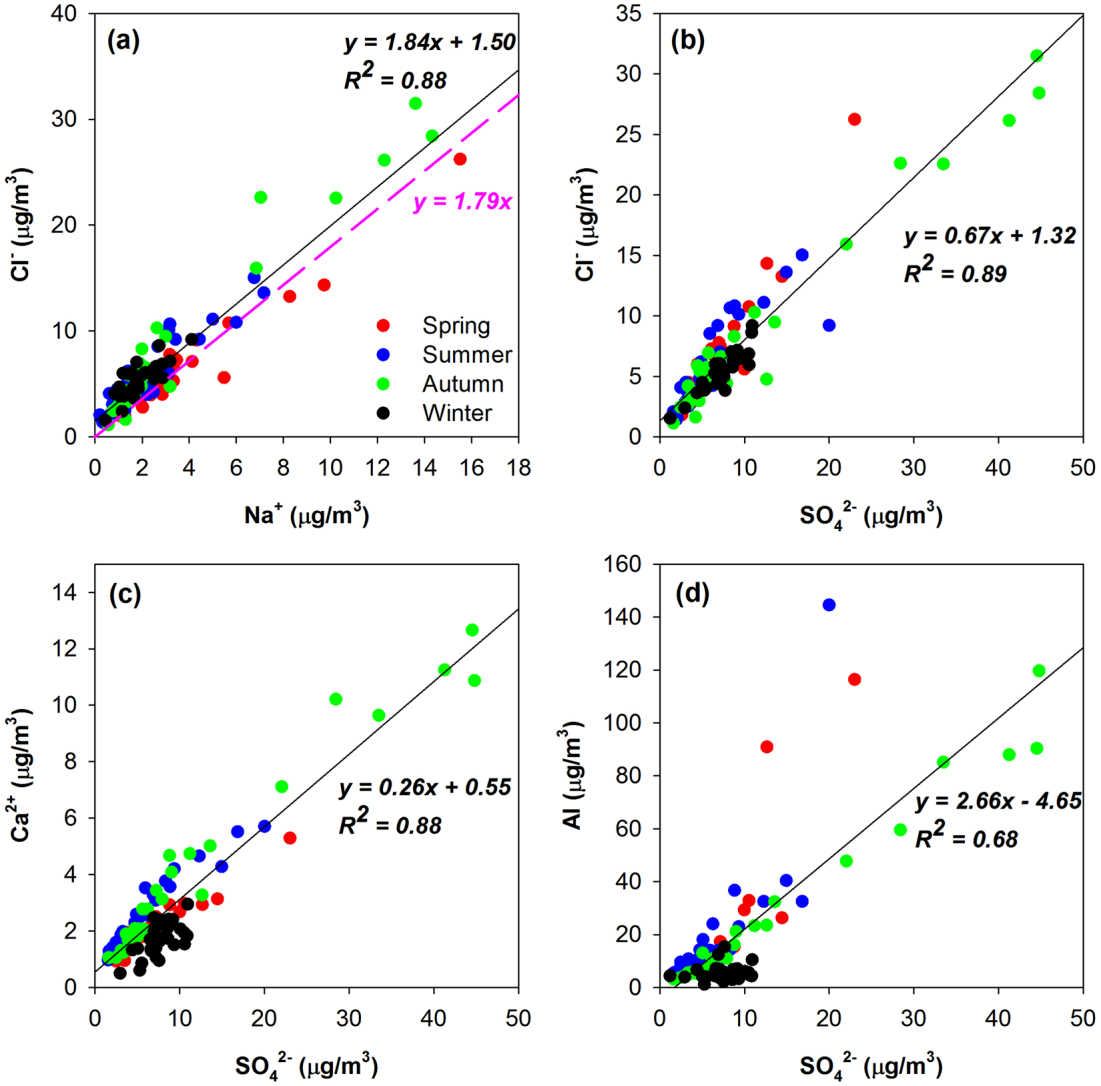
The relationship between (**a**) Cl^−^ and Na^+^, (**b**) Cl^−^ and SO_4_^2−^, (**c**) Ca^2+^ and SO_4_^2−^, and (**d**) Al and SO_4_^2−^. The samples in each season are denoted by different colors. Linear regression (black line) is fitted for the whole study period. The Cl^−^/Na^+^ mass ratio of seawater^34^ is represented by the purple dash line for reference in Fig. 3a.

**Figure 4:**
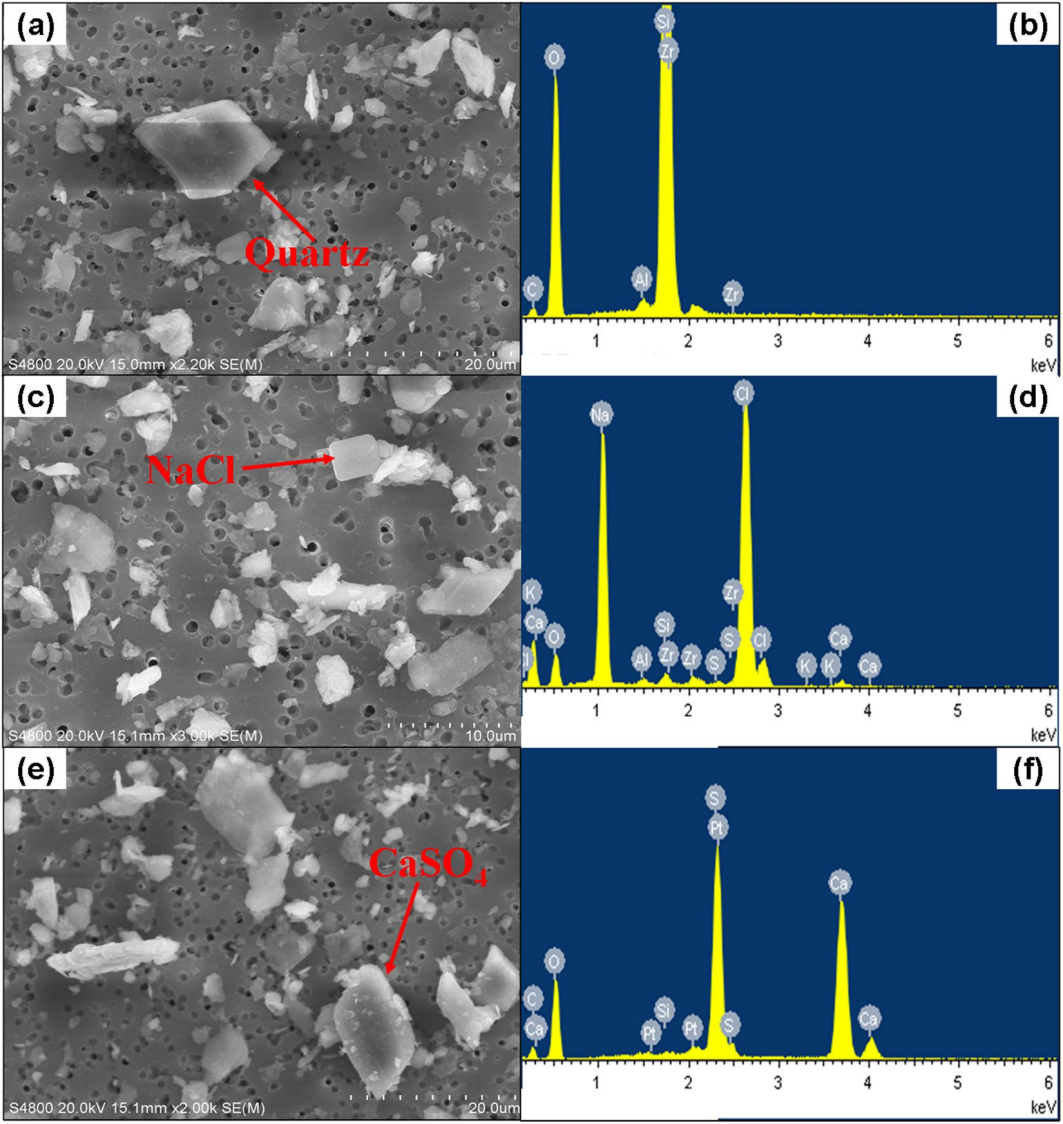
Transmission electron microscopy (TEM) images and energy-dispersive X-ray microanalyzer (EDX) spectra of typical individual components in the Taklimakan dust particles collected in Tazhong (39°N, 83.33°E).

Table 1 shows the average mass concentrations and mass percentages of the measured soluble ions in the particulate matters. We further grouped all the samples into three categories, i.e. DS (dust storm) and NDS (non-dust storm) days during the dust season and NDS days during the non-dust season. Of all the measured ions, SO_4_^2−^, Cl^−^, Na^+^, and Ca^2+^ were the predominant ions, accounting for 84.6% and 90.1% of the total water soluble ions in PM_2.5_ and TSP during the DS days in the dust season. These values varied little of 81.0% and 82.6% during the NDS days in the dust season, indicating the aerosol chemical compositions were relatively stable in the dust season. Compared to the aerosol characteristics of most Chinese urban cities, aerosol over Hotan exhibited unusual features that Cl^−^ and Na^+^ showed considerably high abundances. For instance, during the NDS days in the dust season, Na^+^ and Cl^−^ averaged 0.60 and 1.57 μg/m^3^ in PM_2.5_ and 2.02 and 4.98 μg/m^3^ in TSP, which were at the similar level of their concentrations during the non-dust season. During the DS days in the dust season, the mean concentrations of Na^+^ and Cl^−^ were elevated to 1.13 and 3.27 μg/m^3^ in PM_2.5_ and 8.27 and 17.41 μg/m^3^ in TSP, respectively. The ubiquity of abundant Na^+^ and Cl^−^ throughout all the environmental conditions over Hotan has been rarely observed in the urban, suburban, or even costal areas. As an inland desert area, ocean or crustal sources couldn’t simply explain the very high concentrations of Na^+^ and Cl^−^. Dry salt lakes are ubiquitous over Western China, which are rich in salts. In addition, the Taklimakan Desert was found to be ocean millions years ago^32,33^. In this study, Na^+^ and Cl^−^ exhibited very strong linear correlations in all four seasons as shown in Fig. 3a. The regression slope of Cl^−^ vs. Na^+^ in TSP was fitted as 1.84, fairly close to that of 1.79 in seawater^34^. As for PM_2.5_, Cl^−^ and Na^+^ presented similar feature as TSP with a linear regression Cl^−^/Na^+^ slope of 1.62 (not shown in figure). Individual particle analysis also showed the existence of cubic sea salt particles (Fig. 4c,d). Hence, we believe that the high concentrations of Na^+^ and Cl^−^ over Hotan were strongly related to the dried sea salts from the paleo-ocean in the Taklimakan Desert region in the ancient times. In addition, the widespread dried lakes in the Western China may also have influences on the dust composition^35,36^.

**Table 1:** The mean mass concentrations (μg m^−3^) and percentages (%) of ions in TSP and PM_2.5_ during DS (dust storm) and NDS (non-dust storm) days in the dust and non-dust seasons.

	TSP						PM_2.5_					
	Dust Season				Non-dust Season		Dust season				Non-dust Season	
	DS		NDS		NDS		DS		NDS		NDS	
	Conc.	Percentage	Conc.	Percentage	Conc.	Percentage	Conc.	Percentage	Conc.	Percentage	Conc.	Percentage
SO_4_^2−^	22.98	2.07	5.20	2.73	7.43	6.41	4.15	3.28	1.92	3.93	1.21	11.90
Cl^−^	17.41	1.62	4.98	2.64	5.48	4.84	3.27	2.49	1.57	3.23	0.76	7.04
Na^+^	8.27	0.75	2.02	1.04	1.94	1.63	1.13	0.91	0.60	1.15	0.35	3.43
Ca^2+^	6.71	0.64	2.15	1.15	1.63	1.27	1.95	1.58	0.78	1.50	0.28	2.57
F^−^	0.24	0.03	0.12	0.06	0.07	0.07	0.09	0.06	0.04	0.09	0.01	0.14
NH_4_^+^	1.66	0.17	0.98	0.56	2.33	2.14	0.57	0.39	0.44	1.08	0.61	6.96
K^+^	1.07	0.10	0.46	0.26	0.73	0.62	0.25	0.18	0.19	0.43	0.10	1.10
Mg^2+^	0.44	0.04	0.07	0.03	0.06	0.05	0.03	0.01	—	—	0.01	0.03
NO_3_^−^	2.67	0.26	1.37	0.79	4.09	3.54	0.78	0.48	0.44	0.99	0.65	6.28

#### High calcium dust

Table S2 shows the measured elements under DS and NDS days. It could be clearly seen that the concentrations of all elements were much higher during DS than during ND. However, the contributions of major abundant elements to the particulate masses were fairly close between TSP and PM_2.5_. For instance, the average mass percentage of Al in TSP and PM_2.5_ during DS was 5.80% and 6.03%, respectively. Those ratios were 7.87% and 7.99% for Ca, 4.44% and 5.72% for Fe, 1.97% and 1.88% for K, 2.27% and 2.72% for Mg, 1.89% and 2.11% for Na, 0.33% and 0.37% for Ti, respectively. This indicated that the major abundant elements in the Taklimakan dust aerosol were relatively stable in terms of aerosol size distribution. Ca ranked the highest among the six dust-derived elements (i.e. Al, Ca, Fe, Mg, Mn, and Ti) under all the circumstances as shown in Table S2. The average concentration of Ca in TSP during DS reached 97.60 μg m^−3^ with the peak value of 217.85 μg m^−3^ observed on a super dust storm day (April 16, 2013). Even in NDS, Ca averaged 12.72 μg m^−3^. The mean concentration of Ca in PM_2.5_ was 11.66 and 2.79 μg m^−3^ during DS and NDS, respectively, still higher than any other elements in the fine particles. It was calculated that the mean mass percentage of Ca in TSP and PM_2.5_ reached 7.87% and 7.99% during DS, and 7.42% and 7.44% during NDS, respectively, largely exceeding the abundance of Ca in the natural crust (4.25%). This indicated that dust aerosol over Hotan was characterized of highly enriched Ca, which can be used as a tracer to identify the source of dust originating from the Taklimakan Desert. Table S3 shows the ratios of Ca/Al, Ca/Fe, and Ca/Ti over Hotan. Compared to the Ca/Al ratio of 1.56 over Tazhong, the hinterland of the Taklimakan Desert^1^, Hotan showed very close values. This indicated mineral components of aerosol over Hotan showed very similar characteristics as the Taklimakan Desert and this was expected. Table S3 also shows those ratios over Duolun, a background site in the Gobi Desert^1^, for comparison. It was clearly found that the ratios of Ca/Al, Ca/Fe, and Ca/Ti over Hotan were about 2–3 times of those of Duolun, indicating that these ratios can be regarded as suitable tracers to distinguish the source of dust aerosol originating from these two different desert regions. For instance, during the dust events invading Korea and Japan^37,38^, the Ca/Al and Ca/Ti ratios of the Chinese dust source regions in this study compared well with those ratios observed over the downwind regions. Furthermore, the ratios of Ca/Al and Ca/Ti of the Chinese dust aerosol were found also different from those reported for the Saharan dust^39,40^. A combination of the ratios of Ca/Al (1.5–1.6 of the Taklimakan Desert, 0.6–0.8 of the Gobi Desert, and 1.1–1.4 of the Sahara Desert) and Ca/Ti (25.7–26.8 of the Taklimakan Desert, 9.6–11.7 of the Gobi Desert, and 11.7–14.1 of the Sahara dust) were recommended to be taken as useful tracers to quickly distinguish the dust aerosol from China and Africa.

#### High sulfate from primary origins

As mentioned above, SO_4_^2−^, Cl^−^, Na^+^, and Ca^2+^ were the predominant ions in aerosol throughout the whole year no matter during the dust seasons or non-dust season, while the percentages of other ions increased significantly during the non-dust season. As a comparison, the contribution of SO_4_^2−^, Cl^−^, Na^+^, and Ca^2+^ to the total soluble ions during the non-dust season was much lower with the value of 59.6% and 69.3% in PM_2.5_ and TSP, respectively, compared to that of 84.6% and 90.1% in PM_2.5_ and TSP during the dust season. This was due to that the contribution from NO_3_^−^ and NH_4_^+^ to the total ions increased during the non-dust season when dust activities were much weaker along with the enhanced combustion activities such as residential heating. As an abundant aerosol species especially in the urban environment, the concentration of NO_3_^−^ over Hotan was not rich and much lower than SO_4_^2−^, especially during the dust season (Table 1). As a result, the NO_3_^−^/SO_4_^2−^ ratio exhibited the lowest values of 0.12 and 0.19 in TSP and PM_2.5_ during the DS days in the dust season. These values moderately increased to 0.26 and 0.23 during the NDS days in the dust season. While during the NDS days in the non-dust season, the NO_3_^−^/SO_4_^2−^ ratio reached the highest of 0.55 and 0.54 in TSP and PM_2.5_, respectively. The significant discrepancy of the NO_3_^−^/SO_4_^2−^ ratios under the dust and non-dust conditions implied that the sources of the acidic components in aerosol over Hotan were quite distinct in different seasons.

An obvious feature of the dust aerosol over Hotan was its high concentration of SO_4_^2−^. During the dust seasons, the average concentrations of SO_4_^2−^ in TSP and PM_2.5_ reached as high as 23.0 and 4.2 μg m^−3^, while much lower of 5.2 and 1.9 μg m^−3^ during the non-dust season. Although the mass concentration of SO_4_^2−^ in TSP and PM_2.5_ during DS were about 4.4 and 2.2 times of those during NDS, the contribution of SO_4_^2−^ to the total mass of TSP and PM_2.5_ varied insignificantly with the average mass percentage of 2.73% and 2.07% in TSP during NDS and DS, and 3.93% and 3.28% in PM_2.5_ during NDS and DS, respectively. This probably indicated SO_4_^2−^ was a stable component in the dust particles of the Taklimakan Desert. We further investigated the relationship between SO_4_^2−^ and some typical aerosol components in TSP as shown in Fig. 3. SO_4_^2−^ and Cl^−^ were found highly correlated, yielding the Pearson correlation coefficients (*R*^2^) of 0.89 during the whole study period (Fig. 3b). In addition, there was a strong correlation between Ca^2+^ and SO_4_^2−^ with the *R*^2^ value of 0.88 (Fig. 3c). More interestingly, the element Al, a typical tracer of mineral dust, showed strong correlation with SO_4_^2−^ (Fig. 3d). Similarly, some other mineral dust tracers such as Ti, Fe, and Ca also showed strong correlations with SO_4_^2−^ (not shown in figures). In PM_2.5_, SO_4_^2−^ also presented strong correlations with Cl^−^, Ca^2+^, and Al, yielding the *R*^2^ values of 0.72, 0.73, and 0.84, respectively. This indicated that both fine and coarse mode sulfate were characterized of the same sources. Additional individual particle analysis showed gypsum (CaSO_4_) with regular shapes (Fig. 4e–f). All the evidences above implied that sulfate over Hotan had significant primary sources. From the perspective of paleogeology, the Taklimakan Desert was found to be ocean million years ago^32^. The dried sea salts from the paleo-ocean and the erosion of rocks should be the major sources of sulfate over Hotan.

During the non-dust season (i.e. winter), the correlation coefficients decreased between SO_4_^2−^ and those primary aerosol species while instead increased significantly between SO_4_^2−^ and secondary species (NH_4_^+^, NO_3_^−^), indicating SO_4_^2−^ could be considerably impacted by anthropogenic sources in the non-dust season.

Based on the available literatures, the sulfate mass percentage in the relatively pure dust was around 1.2% along the northeastern rim of the Tengger Desert from another important Asian dust source region, i.e. Gobi Desert^41^. One study on the chemical properties of the Saharan dust indicated that the mass percentages of gypsum (i.e. CaSO_4_) ranged from below detection limit to 0.9% from four major dust source regions in Western Africa^42^. Compared to those studies, our results showed much higher sulfate mass percentages in the Taklimakan dust.

**Figure 5:**
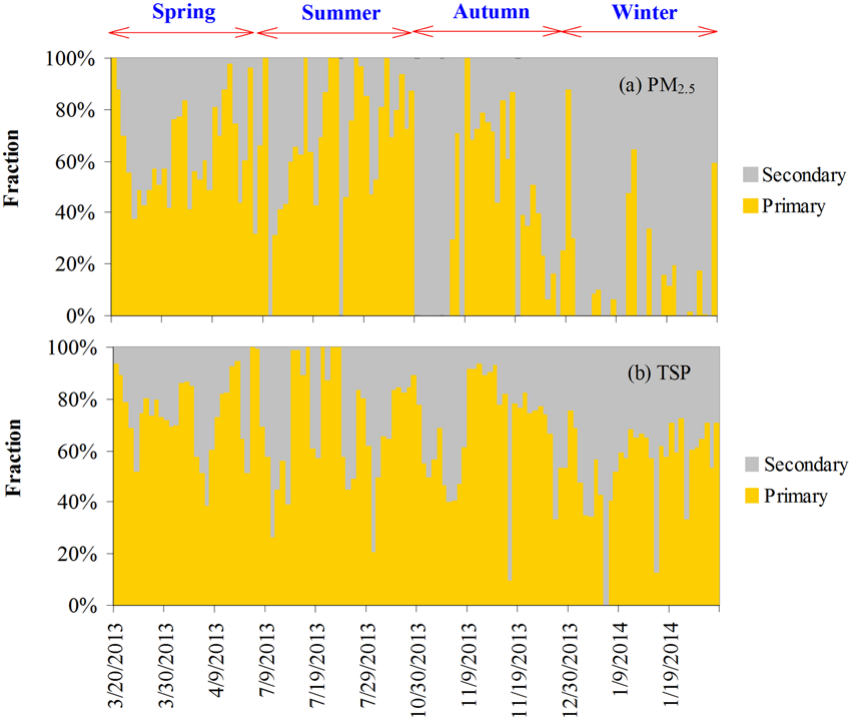
Quantitative assessment of contribution of primary and secondary sources to the total SO_4_^2−^ in TSP and PM_2.5_ over Hotan. No PM_2.5_ data were available during the period of October 30–5 November, 2013.

To distinguish SO_4_^2−^ from primary and anthropogenic sources, we assume that NH_4_^+^ was the only species that neutralized anthropogenic SO_4_^2−^ and NO_3_^−^. As NO_3_^−^ could be regarded as exclusively deriving from anthropogenic sources, the surplus NH_4_^+^ after fully neutralizing NO_3_^−^ was believed to combine with the anthropogenic SO_4_^2−^, i.e. [SO_4_^2−^]_anthropogenic_ = ([NH_4_^+^]/18 – [NO_3_^−^]/62)*48. Then, the percentage of primary and secondary SO_4_^2−^ in the total SO_4_^2−^ can be calculated as *P*[SO_4_^2−^]_anthropogenic_ = [SO_4_^2−^]_anthropogenic_/[SO_4_^2−^]_total_, *P*[SO_4_^2−^]_primary_ = 1 − *P*[SO_4_^2−^]_anthropogenic_. Figure 5 shows the time-series of the percentage of primary and secondary SO_4_^2−^ in the total SO_4_^2−^ in TSP and PM_2.5_, respectively. The mean contribution of primary sources to SO_4_^2−^ during dust seasons was estimated to be (71 ± 20)% and (59 ± 29)% in TSP and PM_2.5_, respectively. During the non-dust season, this contribution was lower of (55 ± 17)% and (14 ± 23)% in TSP and PM_2.5_, respectively. Overall, from the perspective of aerosol chemistry, the high sulfate observed in the dust aerosol from the Taklimakan Desert was characterized of significant primary sources.

## Conclusions

Hotan, a rural site located at the southern edge of the Taklimakan Desert, has been identified as one of the most serious dust pollution regions in China. More than one third of the sampling days exceeded the TSP daily concentrations of 500 μg m^−3^. The frequencies of dust storm over Hotan were the highest in spring, summer, and autumn while the lowest in winter.

Among the soluble ions, SO_4_^2−^, Cl^−^, Na^+^, and Ca^2+^ were the predominant species while prominent ions in the urban environment such as NO_3_^−^ and NH_4_^+^ showed much lower abundances. As a result, the NO_3_^−^/SO_4_^2−^ ratio was around 0.5 during the non-dust season and even lower of less than 0.3 during the dust seasons. Cl^−^ and Na^+^ were observed to have significant abundances in the dust aerosol and these two species showed very strong correlation (R^2^ > 0.9) with the mean Cl^−^/Na^+^ ratio close to that of seawater, which was quite different from other deserts in China. In addition, the content of the elemental Ca in the Taklimakan dust reached higher than 7%, almost two times of that in the natural crust. The Ca/Al ratio is found the highest among the major deserts in the world. At the same time, the soluble part of elemental Ca, i.e. Ca^2+^ presented strong correlations with sulfate. Sulfate also showed significant correlations with typical mineral tracers such as Al, Fe, and Ti as well as Na^+^ and Cl^−^ especially during the dust seasons but almost no relationship with NO_3_^−^, suggesting the high sulfate in the Taklimakan dust aerosol was dominated by non-anthropogenic origins. It was simply estimated that primary sulfate contributed a dominant fraction to the total sulfate during the dust seasons (71 ± 20% and 59 ± 29% in TSP and PM_2.5_) while lower during winter (55 ± 17% and 14 ± 23% in TSP and PM_2.5_).

From the perspective of paleogeology, the Taklimakan Desert was an ocean. In this study, we confirmed that the dust aerosol from the Taklimakan Desert was characterized of strong paelo-oceanic signature. Since the dust aerosol from the Taklimakan Desert contained stable climate forcers such as strong cooling aerosols, e.g. sulfate, future climate modeling should consider the explicit composition of dust aerosol as the long-range transport of dust could have profound climatic impacts on the continental or even global scales. In addition, preliminary results on individual dust particle analysis suggested sulfate mainly externally mixed with other components, which was quite different from urban environment where sulfate was mostly internally mixed (e.g. coating on black carbon). However, more analytical techniques such as XRD (X-ray diffraction) should be applied to further characterize the chemical forms of the dust aerosol. Overall, this study suggested that climate modeling should also consider the mixing states of dust from the Taklimakan Desert as different mixing states could result in distinct climatic impacts.

## Electronic supplementary material

Supplementary Materials
