# Supplementary material for: First long-term detection of paleo-oceanic signature of dust aerosol at the southern marginal area of the Taklimakan Desert
Source: Sci Rep. 2018 Apr 30;8:6779. doi: 10.1038/s41598-018-25166-5 (PMC5928038; doi:10.1038/s41598-018-25166-5)
Supplement: Supplementary file 1 — Supplementary Materials [file 41598_2018_25166_MOESM1_ESM.doc]

**Supplementary Materials**

**First long-term detection of paleo-oceanic signature of dust aerosol at** **the southern marginal area of the Taklimakan Desert**

Qi Zhou^1,#^, Juan Li^1,#^, Jian Xu^1^, Xiaofei Qin^1^, Congrui Deng^1^, Joshua S. Fu^3^, Qiongzhen Wang^4^, Mijiti Yiming^5^, Kan Huang^1,2,*^, Guoshun Zhuang^1,*^

^1^Center for Atmospheric Chemistry Study, Shanghai Key Laboratory of Atmospheric Particle Pollution and Prevention (LAP3), Department of Environmental Science and Engineering, Fudan University, Shanghai 200433, China

^2^Institute of Atmospheric Sciences, Fudan University, Shanghai 200433, China

^3^Department of Civil and Environmental Engineering, University of Tennessee, Knoxville, TN 37996, USA

^4^Environmental Science Research & Design Institute of Zhejiang Province, Hangzhou, Zhejiang 310007, China

^5^Hetian Environmental Monitoring Center, Hetian, 848000, China

# Co-first author

* Correspondence: huangkan@fudan.edu.cn; gzhuang@fudan.edu.cn

Table S1. The concentration (μg m^-3^) of TSP and PM_2.5_ over major desert regions in China (one standard deviation is shown in the [bracket](http://www.baidu.com/link?url=8Qbq1mEQOaTfdv_Mfucwd_klPgdoRDx5mPXahhxset7zuFuhgQlDkXJaIC6Rg8edCgQHQIOIP24wyRN49l00iqpYRLGCtsscXN_KUKi5KdMxo4AF8Q5a8wKDLxBrdyJk))

| Sites | Time | TSP | PM_2.5_ | PM_2.5_/TSP | Reference |
| --- | --- | --- | --- | --- | --- |
| Hotan | 2011-2013 | 405.3 (578.0) | 66.6 (58.9) | 0.25 (0.18) | This work |
| Yulin | 2007-2008 (spring) | 189.4 | 59.7 | 0.31 |  |
| Duolun | 2013 | 107.5 | 46.2 | 0.43 | Unpublished data |

Table S2. The concentration (μg m^-3^) and the percentage(%) of elements in TSP and PM_2.5_ on DS and NDS during dust seasons

|  | TSP | | | | | | |  | PM_2.5_ | | | | | | |
| --- | --- | --- | --- | --- | --- | --- | --- | --- | --- | --- | --- | --- | --- | --- | --- |
|  | DS | |  | ND | |  | DS/ND |  | DS | |  | ND | |  | DS/ND |
|  | Conc. | percentage |  | Conc. | percentage |  | Ratio |  | Conc. | percentage |  | Conc. | percentage |  | Ratio |
| Particles | 1425.23 |  |  | 202.10 |  |  |  |  | 146.33 |  |  | 50.90 |  |  |  |
| Al | 74.06 | 5.80 |  | 10.14 | 5.08 |  | 6.87 |  | 8.64 | 6.03 |  | 2.59 | 5.16 |  | 3.34 |
| As | 0.02 | 0.00 |  | 0.00 | 0.00 |  | 5.05 |  | 0.01 | 0.00 |  | 0.00 | 0.01 |  | 1.30 |
| Ba | 0.53 | 0.04 |  | 0.07 | 0.04 |  | 7.17 |  | 0.06 | 0.04 |  | 0.02 | 0.04 |  | 2.77 |
| Ca | 97.60 | 7.87 |  | 14.65 | 7.42 |  | 6.66 |  | 11.66 | 7.99 |  | 3.69 | 7.44 |  | 3.16 |
| Cd | 0.01 | 0.00 |  | 0.00 | 0.00 |  | 3.65 |  | 0.00 | 0.00 |  | 0.00 | 0.00 |  | 1.54 |
| Co | 0.01 | 0.00 |  | 0.00 | oji |  | 4.26 |  | 0.00 | 0.00 |  | 0.00 | 0.00 |  | 3.10 |
| Cr | 0.07 | 0.01 |  | 0.02 | 0.01 |  | 3.09 |  | 0.02 | 0.01 |  | 0.01 | 0.02 |  | 1.69 |
| Cu | 0.08 | 0.01 |  | 0.04 | 0.02 |  | 1.90 |  | 0.03 | 0.02 |  | 0.02 | 0.08 |  | 1.23 |
| Fe | 57.31 | 4.44 |  | 8.87 | 4.49 |  | 6.46 |  | 8.29 | 5.72 |  | 2.56 | 5.60 |  | 3.24 |
| K | 26.96 | 1.97 |  | 3.43 | 1.72 |  | 7.87 |  | 2.75 | 1.88 |  | 0.84 | 1.71 |  | 3.30 |
| Mg | 25.92 | 2.27 |  | 4.30 | 2.16 |  | 6.03 |  | 3.87 | 2.72 |  | 1.19 | 2.37 |  | 3.26 |
| Mn | 0.82 | 0.06 |  | 0.12 | 0.06 |  | 6.81 |  | 0.11 | 0.08 |  | 0.03 | 0.07 |  | 3.20 |
| Na | 20.97 | 1.89 |  | 4.24 | 2.15 |  | 4.94 |  | 2.85 | 2.11 |  | 1.25 | 2.54 |  | 2.27 |
| Ni | 0.05 | 0.00 |  | 0.01 | 0.01 |  | 4.35 |  | 0.01 | 0.00 |  | 0.01 | 0.05 |  | 0.70 |
| P | 0.65 | 0.04 |  | 0.09 | 0.04 |  | 7.53 |  | 0.06 | 0.04 |  | 0.02 | 0.04 |  | 2.94 |
| Pb | 0.05 | 0.01 |  | 0.04 | 0.02 |  | 1.40 |  | 0.02 | 0.02 |  | 0.02 | 0.04 |  | 1.48 |
| S | 6.47 | 0.52 |  | 1.31 | 0.70 |  | 4.95 |  | 1.09 | 0.79 |  | 0.44 | 0.95 |  | 2.47 |
| Sn | 0.01 | 0.00 |  | 0.00 | 0.00 |  | 3.60 |  | 0.00 | 0.00 |  | 0.00 | 0.01 |  | 1.02 |
| Sr | 0.46 | 0.04 |  | 0.07 | 0.04 |  | 6.37 |  | 0.07 | 0.05 |  | 0.02 | 0.04 |  | 3.14 |
| Ti | 4.76 | 0.33 |  | 0.65 | 0.33 |  | 7.37 |  | 0.53 | 0.37 |  | 0.17 | 0.33 |  | 3.20 |
| Zn | 0.18 | 0.01 |  | 0.08 | 0.05 |  | 2.19 |  | 0.06 | 0.04 |  | 0.04 | 0.10 |  | 1.40 |
| Mineral | 1104.60 | 84.14 |  | 155.81 | 78.22 |  | 7.09 |  | 133.19 | 85.10 |  | 40.42 | 75.97 |  | 3.30 |

Table S3. Typical elemental ratios for TSP over Hotan and Duolun (Gobi Desert)

| TSP | Hotan | | | |  | Duolun | | | |
| --- | --- | --- | --- | --- | --- | --- | --- | --- | --- |
|  | Spring | Summer | Autumn | Winter |  | Spring | Summer | Autumn | Winter |
| Ca/Al | 1.58±0.10 | 1.54±0.07 | 1.63±0.11 | 1.59±0.15 |  | 0.61±0.12 | 0.88±0.20 | 0.84±0.22 | 0.78±0.12 |
| Ca/Fe | 2.46±0.14 | 2.35±0.17 | 2.32±0.15 | 2.34±0.20 |  | 1.18±0.10 | 1.36±0.20 | 1.77±0.18 | 1.45±0.12 |
| Ca/Ti | 25.08±1.60 | 24.65±1.42 | 26.75±1.47 | 25.60±1.57 |  | 9.57±1.27 | 11.72±1.65 | 11.00±2.27 | 10.12±1.31 |
